# Unusual Life-Threatening Pneumothorax Complicating a Ruptured Complex Aspergilloma in an Immunocompetent Patient in Cameroon

**DOI:** 10.1155/2018/8648732

**Published:** 2018-02-15

**Authors:** Bernadette Ngo Nonga, Bonaventure Jemea, Angele O. Pondy, Daniel Handy Eone, Marie Claire Bitchong, Olivier Fola, Atems Nkolaka, Gilles Martin Londji

**Affiliations:** ^1^Department of Surgery, University Hospital Centre of Yaoundé, University of Yaoundé I, Yaounde, Cameroon; ^2^Department of Anesthesia and Intensive Care Unit, University Hospital Centre of Yaoundé, University of Yaoundé I, Yaounde, Cameroon; ^3^Department of Pediatrics, University of Yaoundé I, Yaounde, Cameroon; ^4^Department of Medicine, Faculty of Medicine and Pharmaceuticals Sciences, University of Douala, Douala, Cameroon

## Abstract

An aspergilloma is a well-recognized lesion of the lung caused most of the time by the fungus *Aspergillus fumigatus*. Its main complication is hemoptysis and has been very rarely associated with tension pneumothorax. We present the case of a 47-year-old man with a history of treated and healed tuberculosis, which was successfully managed in our service for a ruptured right upper lobe complexed aspergilloma, complicated by a massive and tension pneumothorax. The patient underwent thoracotomy and lung resection with quick recovery. Conclusively, although rare, an aspergilloma may rupture and cause a life-threatening air leakage.

## 1. Background

Spontaneous pneumothorax is a common disease. However, its association with pulmonary infection due to *Aspergillus fumigatus* is very rare [1].

Pulmonary aspergillosis is a diffuse infection of the lung which is usually associated with immunodepression secondary to cancer and its treatments, transplantation, human immunodeficiency virus infection, and steroid treatment as used in chronic lung diseases.

Aspergilloma, on the other hand, is the localized form of infection resulting from colonization of a preexisting tuberculosis or lung abscess cavity usually by *Aspergillus fumigatus* [1]. Its primary and main symptom is hemoptysis, and very few cases have been associated with pneumothorax [1, 2].

Sakuraba et al. have reported 11 cases of *Aspergillus* infection discovered after surgery for pneumothorax [3]. However, there was no connection to suggest this association in most of the cases. Nonetheless, in one case, only the *Aspergillus* may have been responsible for the pneumothorax due to the rupture of bullae. Gupta et al. have reported the rupture of a complex aspergilloma associated with a simple pneumothorax; however, the patient died 5 days after admission [1]. Cases of pleura or pulmonary aspergillosis have been associated with pneumothorax, but ruptured complex aspergilloma with tension pneumothorax has not been reported. We are reporting a case of a man with a history of treated and healed tuberculosis, who was referred to us for a spontaneous tension pneumothorax secondary to a ruptured complex aspergilloma cavity.

## 2. Case Presentation

**Figure 1:**
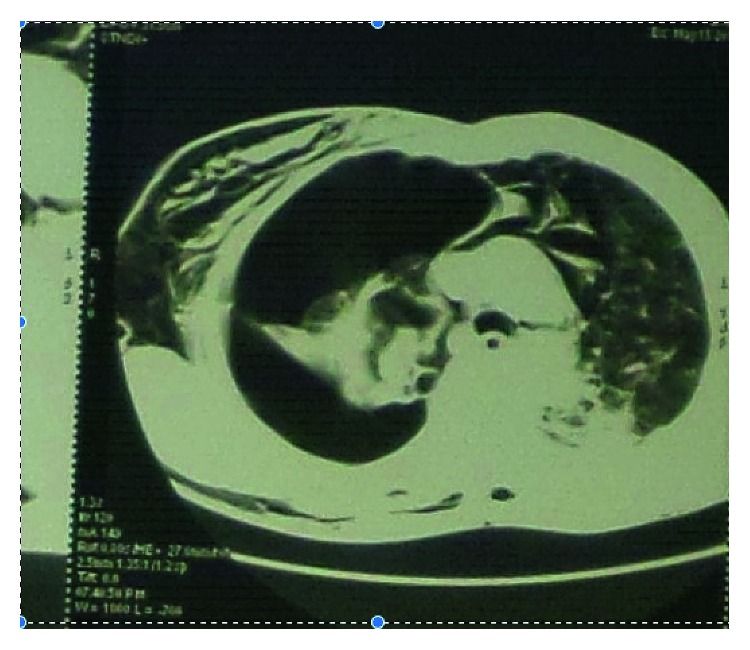
Chest CT scan transverse view. The aspergilloma cavity is seen at the apex of the right lung. There are massive pneumothorax with subcutaneous emphysema and bilateral lung fibrosis as sequelae of tuberculosis.

**Figure 2:**
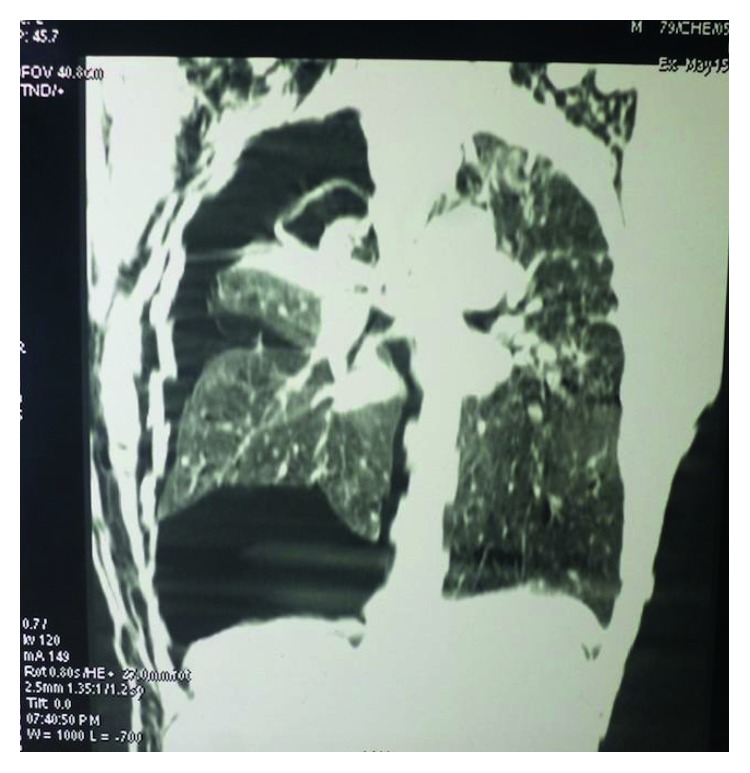
Chest CT scan frontal view. The aspergilloma is seen in the apical region. There is extensive subcutaneous emphysema bilaterally. The upper lobe is totally destroyed, the middle lobe is fibrotic, and the lower lobe is atelectatic and retracted. There are massive pneumothorax, bilateral fibrosis, and sequelae of tuberculosis mainly at the left apex.

The patient was a 47-year-old man, sport teacher, referred to us for a persistent respiratory distress. He was taken to a city hospital 3 days before admission for severe acute respiratory distress and chest pain. A chest X-ray done in that hospital revealed a pneumothorax on the right side, and a chest tube was then inserted. Despite the presence of the indwelling chest tube, the patient continued to complain of dyspnoea and later developed subcutaneous emphysema. A chest CT scan was then requested to look for the possible cause of the pneumothorax. It revealed a massive pneumothorax with subcutaneous emphysema on the right side associated with a typical complex aspergilloma cavity well defined at the right lung apex (Figures 1 and 2). The patient was then referred to our hospital for further management.

On arrival, he was conscious, alert, and oriented in time and space but dyspnoeic, presenting with signs of respiratory distress. The vital parameters on arrival revealed the following: blood pressure: 136/93 mmHg, heart rate: 86 beats/min, respiratory rate: 28 cycles/min, temperature: 38.5°C, and oxygen saturation: 79% on 6 l/min of nasal oxygen. There was massive air in the drain. A second chest drain was inserted. As a sport teacher, he did exercises regularly. He was treated for pulmonary tuberculosis 2 years back and was declared healed. Elsewhere, his past medical and surgical history was unremarkable. He had no specific complaints for 2 years and had been fine ever since. He also complained of cough and blood-stained sputum.

**Figure 3:**
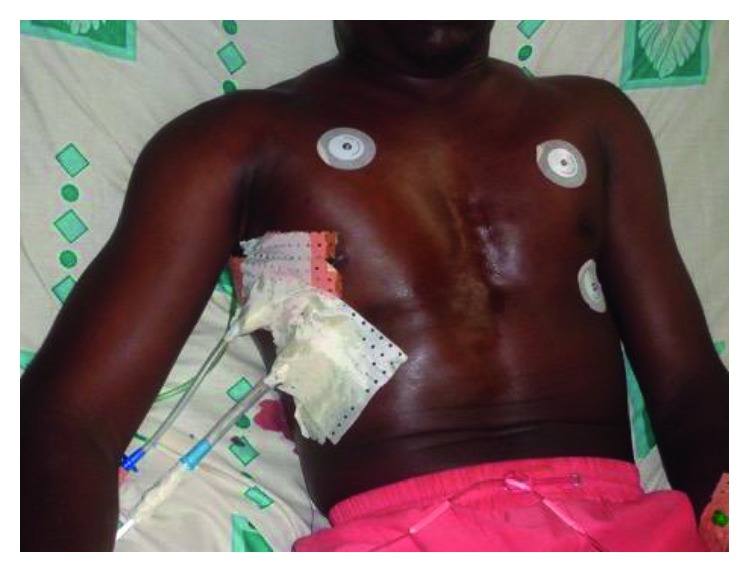
Picture showing the patient with massive subcutaneous emphysema despite 2 chest tubes inserted.

**Figure 4:**
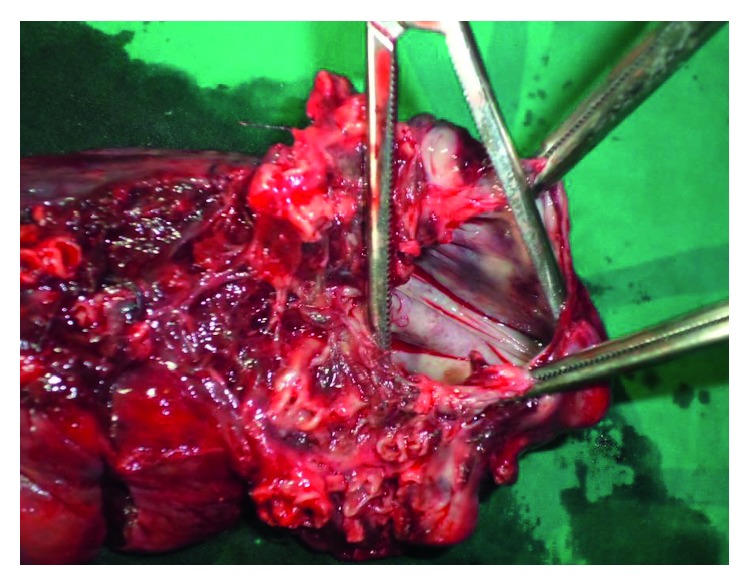
Photo of the specimen. Inside the aspergilloma cavity, a large cavity communicating with the main bronchus is shown, and the aspergilloma truffe has been removed from the cavity.

The physical exam revealed an anxious patient with severe dyspnoea and massive subcutaneous emphysema (diffuse subcutaneous air with tympanic chest, all over the trunk and the cervical and scrotal regions as seen in Figure 3). Despite insertion of the second drain, there was still a persistent significant air leak with hypoxia. A preoperative workup was thus quickly done and revealed a hemoglobin count of 14 g/dl, a negative HIV serology, and a normal coagulation profile. The other laboratory results were normal, and the patient was taken to the operating room for a thoracotomy one day after admission. The patient underwent a right thoracotomy under general anaesthesia with a selective lung intubation obtained by the regular endotracheal tube pushed in the left lung. We carried out a small posterior muscle-sparing incision of 14 cm and entered the chest at the fifth intercostal space as previously reported [4]. Perioperatively, we found massive pleuropulmonary adhesions in the mediastinal region. The upper and middle lobes were totally collapsed, fibrotic, and very small. The lower lobe had less adhesions and was thus freer. A decortication was done with caution. We found chronic pulmonary aspergillosis (aspergilloma cavity containing the fungal ball) in the right upper lobe. The entire right upper lobe had been replaced by the aspergilloma and directly connected to the main bronchus. The middle lobe was fibrotic and diffusely diseased. There were no visible bullae (Figure 4).

**Figure 5:**
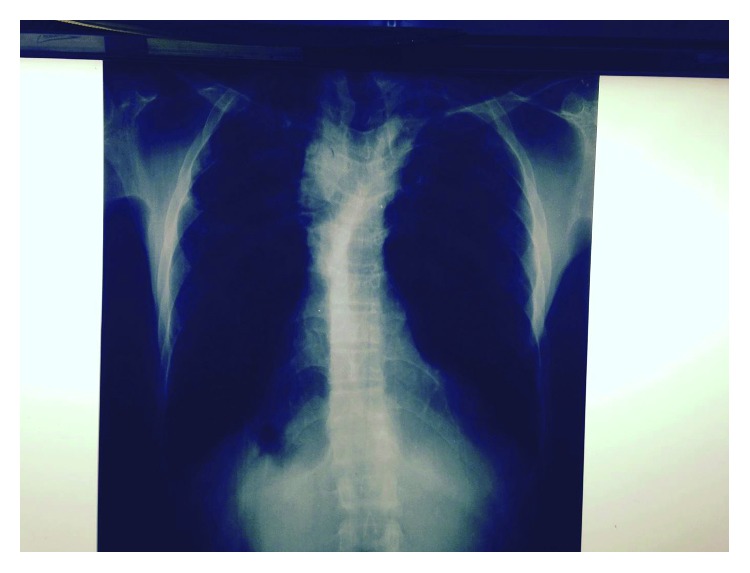
Chest X-ray after 17 months showing a fully expanded lower lobe and the same fibrosis in both lungs with no change.

We did a double lobectomy: upper and middle. There was bleeding of moderate volume due to the decortication. Only one drain was left in place, and the simple drainage system that we have reported was used, which uses respiratory movements and gravity [5]. The drainage system was left open on the first day and converted to a closed system 3 days after the surgery. He received amoxicillin and clavulanic acid as antibiotics for 72 hours, tramadol and diclofenac for pain control, and enoxaparin for prevention of deep venous thrombosis. He resumed regular diet the same day after surgery and was out of bed the next day. Incentive spirometer was done having the patient blows in a rubber glove. The chest tube was removed on post-op day 5, and the patient was discharged 6 days after surgery. The pathology report confirmed the aspergilloma with no signs of active tuberculosis. He was seen as outpatient for regular follow-up and did not receive any more antituberculosis medication because the pathology did not show signs of persistent active disease; this was confirmed on the follow-up chest radiograph 17 months later (Figure 5).

## 3. Discussion

As reported in literature, hemoptysis is the main complication of aspergilloma, and massive pneumothorax is rare [1] especially in patients with tuberculosis where healing is associated with fibrosis and pleurodesis. Complex aspergilloma is usually adherent to the apex of the lung with a thick wall as was the case in our patient. This case is very unusual and surprising because of the history of healed tuberculosis associated with some kind of fibrosis as can be seen in the CT scan of the patient (Figures 1 and 2) even though extremely rare in an immunocompetent patient.

Tuberculosis and HIV/AIDS are common infections in Cameroon. They can be associated in the same patient yet there have not been many cases of spontaneous pneumothorax reported in patients with pulmonary aspergilloma or chronic pulmonary aspergillosis. Gupta et al. have reported the only case with spontaneous rupture of a complex aspergilloma.

Our case is unique because the rupture of the aspergilloma cavity itself resulted in a tension pneumothorax with massive subcutaneous and life-threatening emphysema. Some authors have reported cases of pneumothorax associated with pleural and lung aspergillosis but not with complex aspergilloma [2, 3].

Although the rupture was subpleural, there were no bullae discovered during surgery. Unlike many other spontaneous pneumothoraxes, it was not due to rupture of a bulla but due to the spontaneous rupture of the aspergilloma resulting from the diseased entire upper lobe reduced to a cavity; in this case, the cavity was connected directly to the main bronchus resulting in massive air leak after rupture (Figure 4). Sakuraba et al. [3] have reported 11 cases of pneumothorax associated with *Aspergillus* infection with no direct causal effect.

The mortality associated with a ruptured aspergilloma complicated by pneumothorax has been very high as reported by Gupta et al., in a similar case where the immunocompetent patient died 5 days after admission [1]. Martino et al. reported 6 cases of pneumothorax in 46 immunosuppressed patients after bone marrow transplant, of which four of them died from it [2].

The decision to observe or to treat with an immediate intervention is guided by risk stratification that considers the patient's presentation and the likelihood of spontaneous resolution and recurrence. Although surgery for aspergilloma is very difficult, it is the preferred treatment. The European Respiratory Society recommends to surgically excise simple aspergilloma if technically possible preferably using video-assisted thoracic surgery [6]. In western countries, video-assisted thoracoscopy (VATS) is the preferred treatment [3]. In our environment, VATS is not available, and even so, it would have been very difficult to operate this case under VATS as there was massive air leak. We had to clamp the upper main bronchus before proceeding to the lung dissection, and there were a lot of adhesions on the medial side, which would have rendered endoscopic surgery difficult. This procedure is not even available in our country.

The best treatment for patients with complicated aspergilloma in Cameroon is surgical as the newly active antifungal drugs (itraconazole and voriconazole) are not available in our environment. Our patient underwent a successful resection of the diseased lung with no complication.

## 4. Conclusion

Aspergilloma is rarely associated with pneumothorax. We have reported one of the few cases complicated with an unusual massive pneumothorax which was managed successfully in an immunocompetent patient.

## Additional Points

All data and material are available for verification.
